# Supplementary figures and images for: Overriding impaired FPR chemotaxis signaling in diabetic neutrophil stimulates infection control in murine diabetic wound
Source: eLife. 2022 Feb 3;11:e72071. doi: 10.7554/eLife.72071 (PMC8846594; doi:10.7554/eLife.72071)

## Slide 1
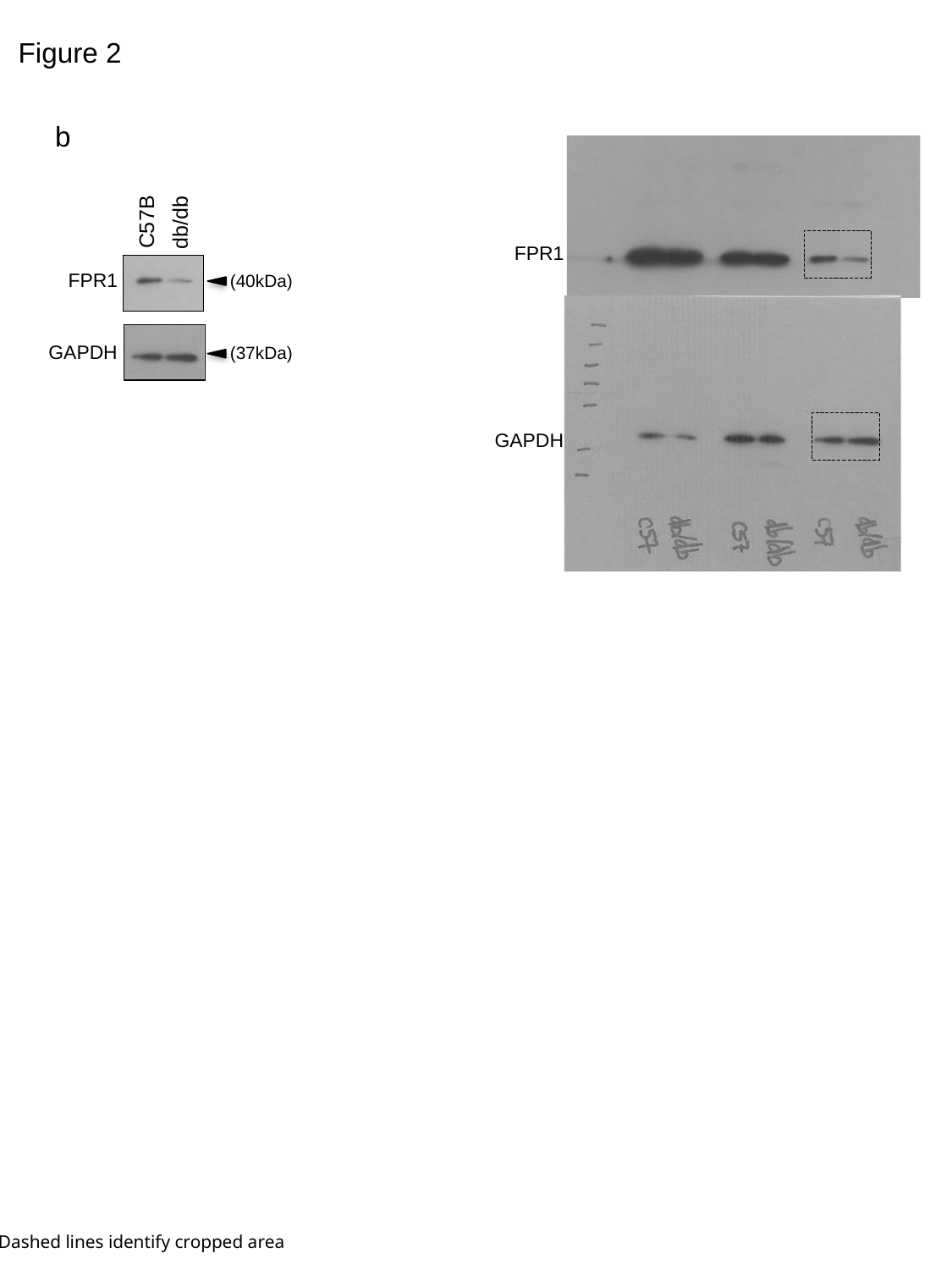

Figure 2
b
C57B
db/db
FPR1
FPR1
(40kDa)
GAPDH
(37kDa)
GAPDH
Dashed lines identify cropped area

Supplement: Figure 2—source data 2. [file elife-72071-fig2-data2.pptx]

## Slide 1
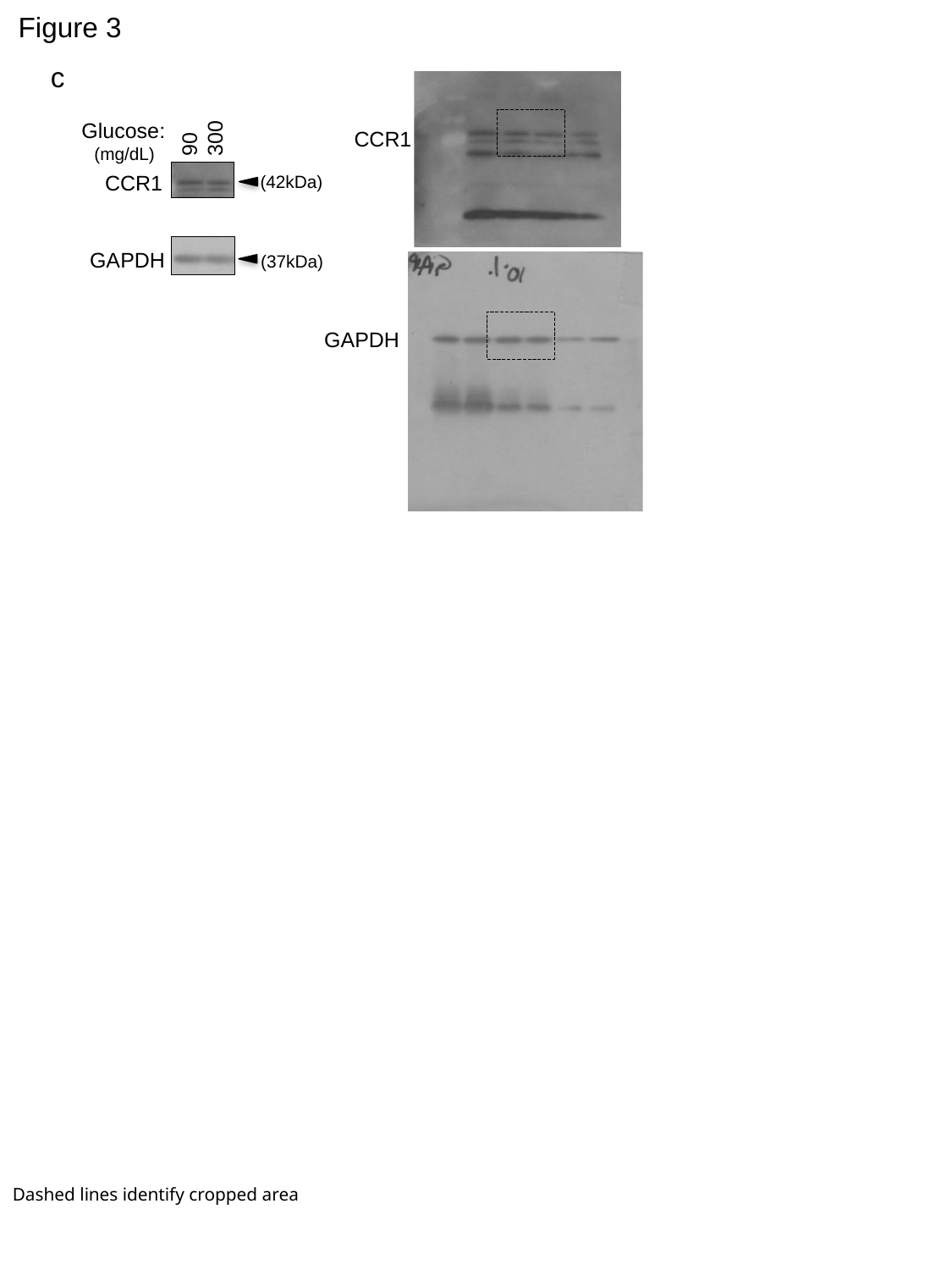

Figure 3
c
Glucose:
(mg/dL)
300
90
CCR1
(42kDa)
GAPDH
(37kDa)
CCR1
GAPDH
Dashed lines identify cropped area

Supplement: Figure 3—source data 3. [file elife-72071-fig3-data3.pptx]

## Slide 1
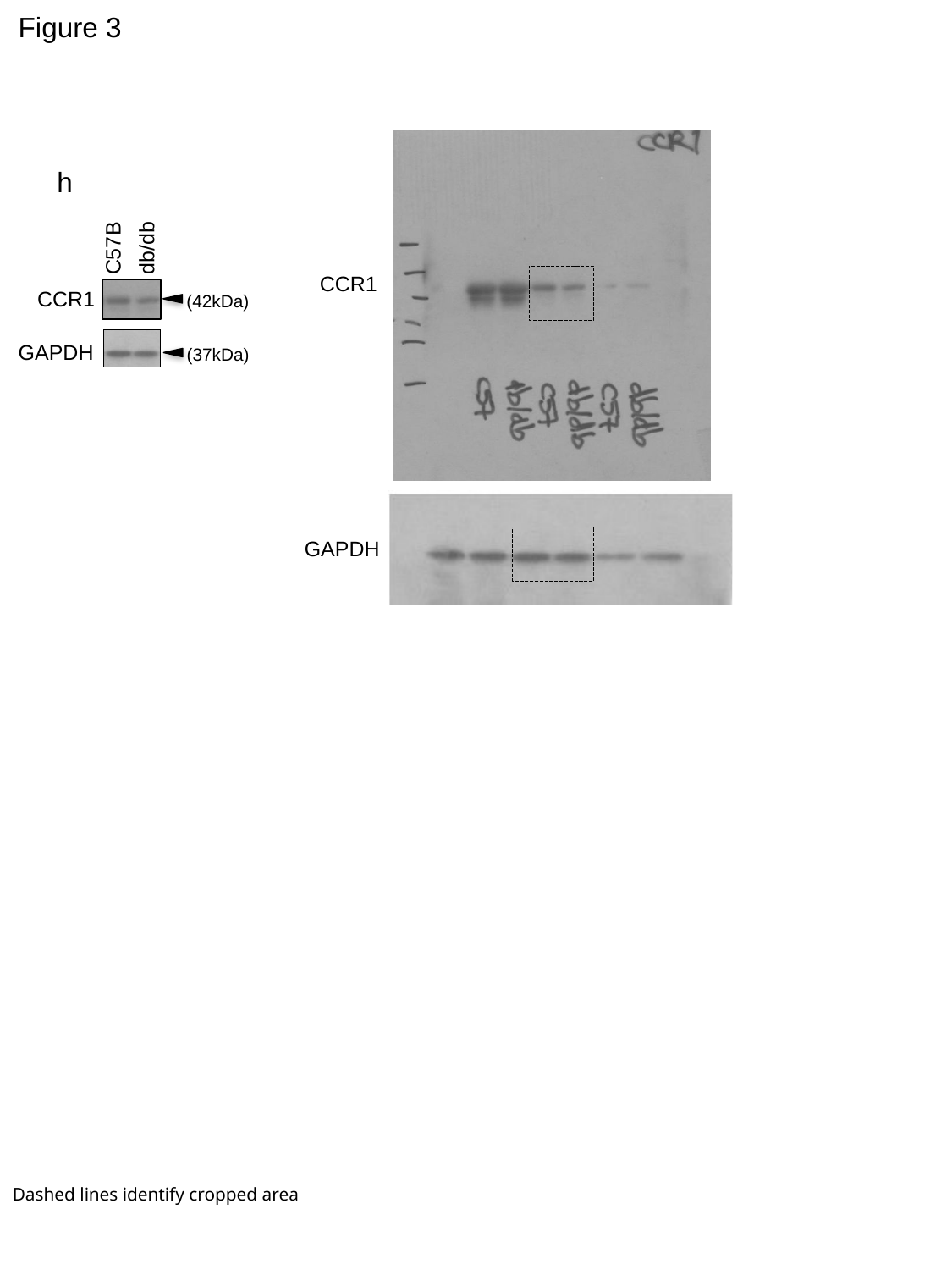

Figure 3
h
db/db
C57B
CCR1
(42kDa)
GAPDH
(37kDa)
CCR1
GAPDH
Dashed lines identify cropped area

Supplement: Figure 3—source data 7. [file elife-72071-fig3-data7.pptx]
