# Supplementary material for: Overriding impaired FPR chemotaxis signaling in diabetic neutrophil stimulates infection control in murine diabetic wound
Source: eLife. 2022 Feb 3;11:e72071. doi: 10.7554/eLife.72071 (PMC8846594; doi:10.7554/eLife.72071)
Supplement: Figure 2—source data 8. [file elife-72071-fig2-data8.pptx]

## Slide 1
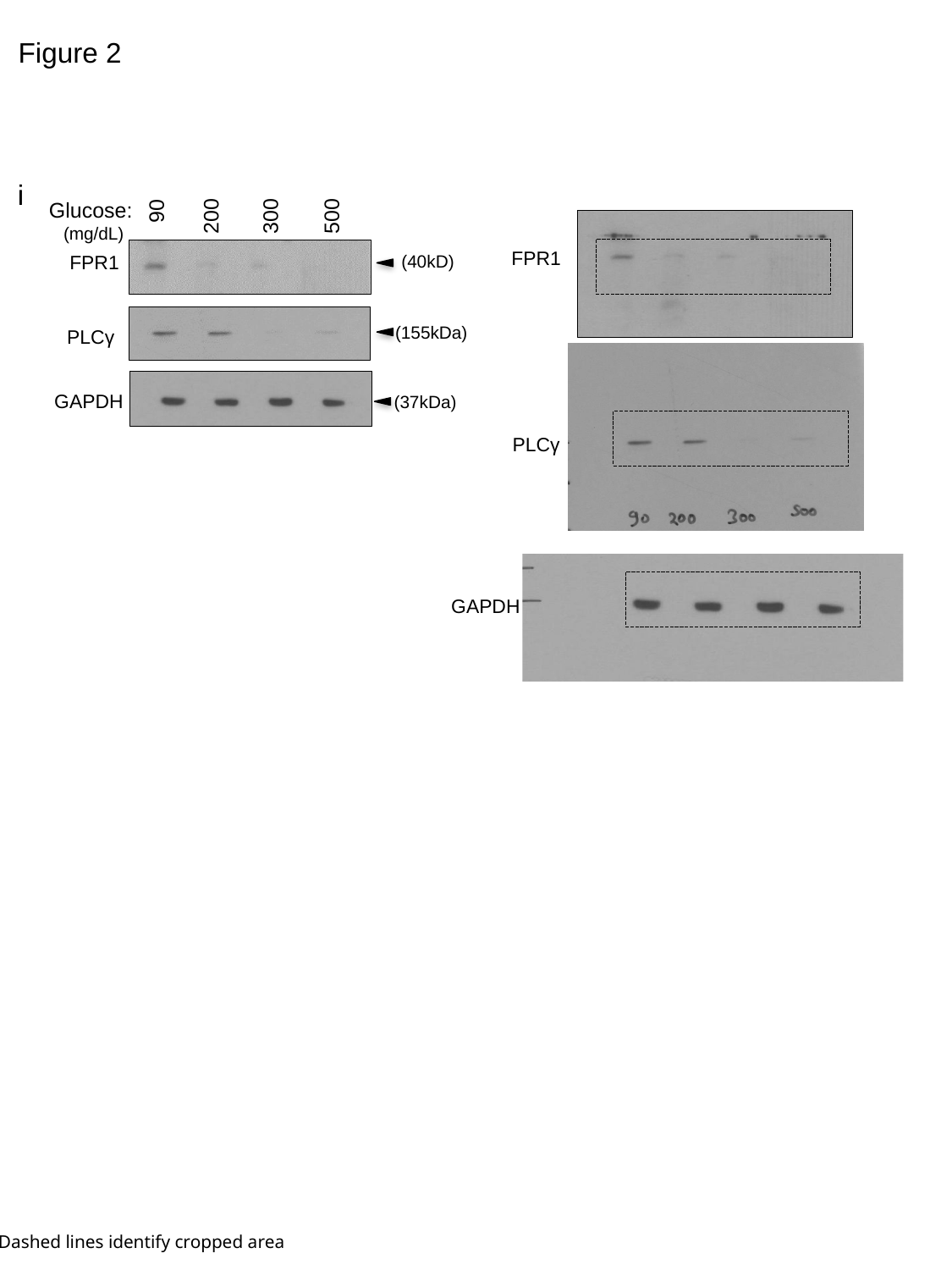

Figure 2
i
Glucose:
 (mg/dL)
90
300
500
200
FPR1
(40kD)
FPR1
(155kDa)
PLCγ
GAPDH
(37kDa)
PLCγ
GAPDH
Dashed lines identify cropped area
