# Supplementary material for: Overriding impaired FPR chemotaxis signaling in diabetic neutrophil stimulates infection control in murine diabetic wound
Source: eLife. 2022 Feb 3;11:e72071. doi: 10.7554/eLife.72071 (PMC8846594; doi:10.7554/eLife.72071)
Supplement: Figure 2—figure supplement 2—source data 2. [file elife-72071-fig2-figsupp2-data2.pptx]

## Slide 1
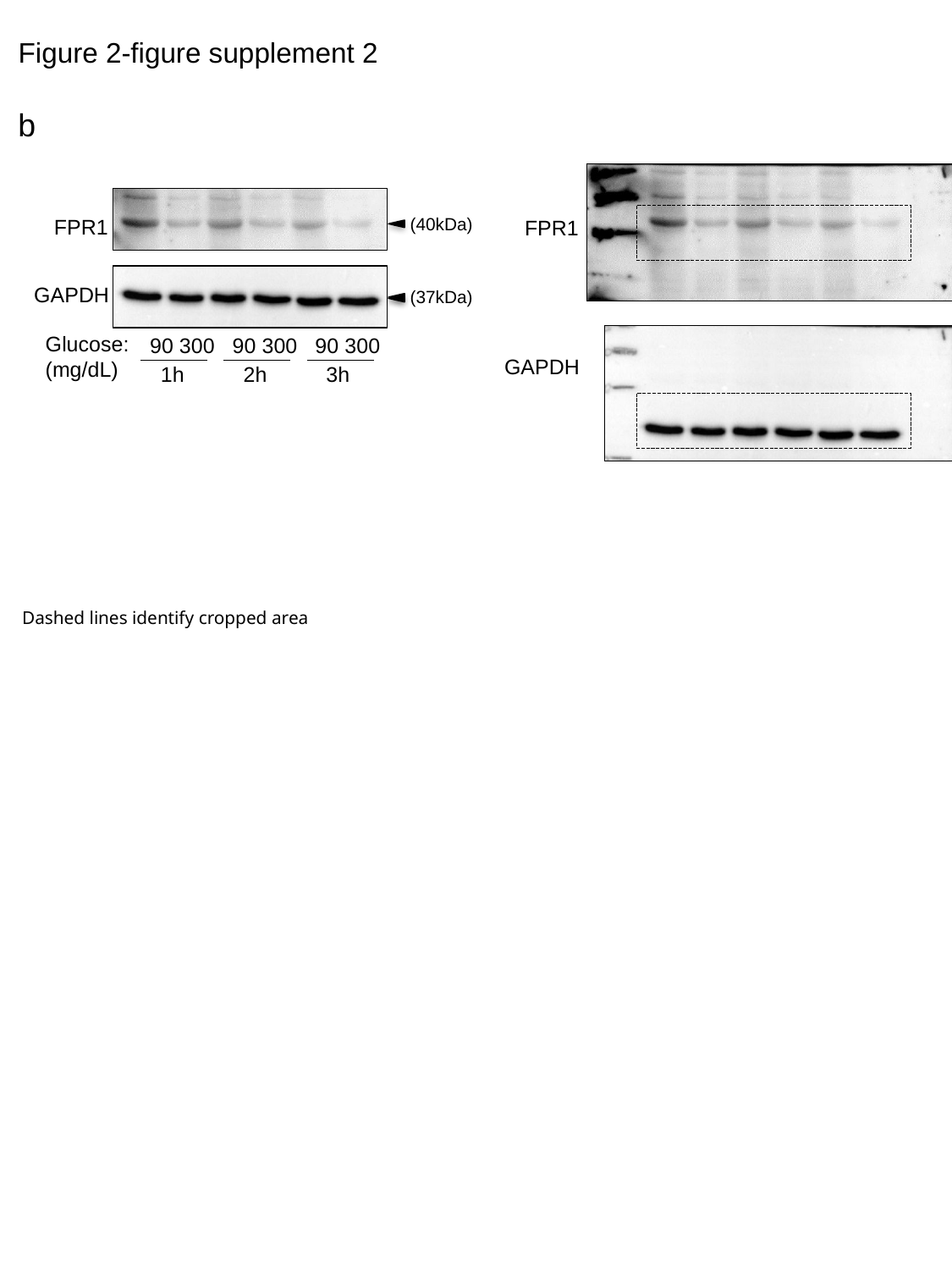

Figure 2-figure supplement 2
b
(40kDa)
FPR1
FPR1
GAPDH
(37kDa)
Glucose:
(mg/dL)
90 300 90 300 90 300
1h 2h 3h
GAPDH
Dashed lines identify cropped area
