# Supplementary material for: Overriding impaired FPR chemotaxis signaling in diabetic neutrophil stimulates infection control in murine diabetic wound
Source: eLife. 2022 Feb 3;11:e72071. doi: 10.7554/eLife.72071 (PMC8846594; doi:10.7554/eLife.72071)
Supplement: Figure 4—source data 2. [file elife-72071-fig4-data2.pptx]

## Slide 1
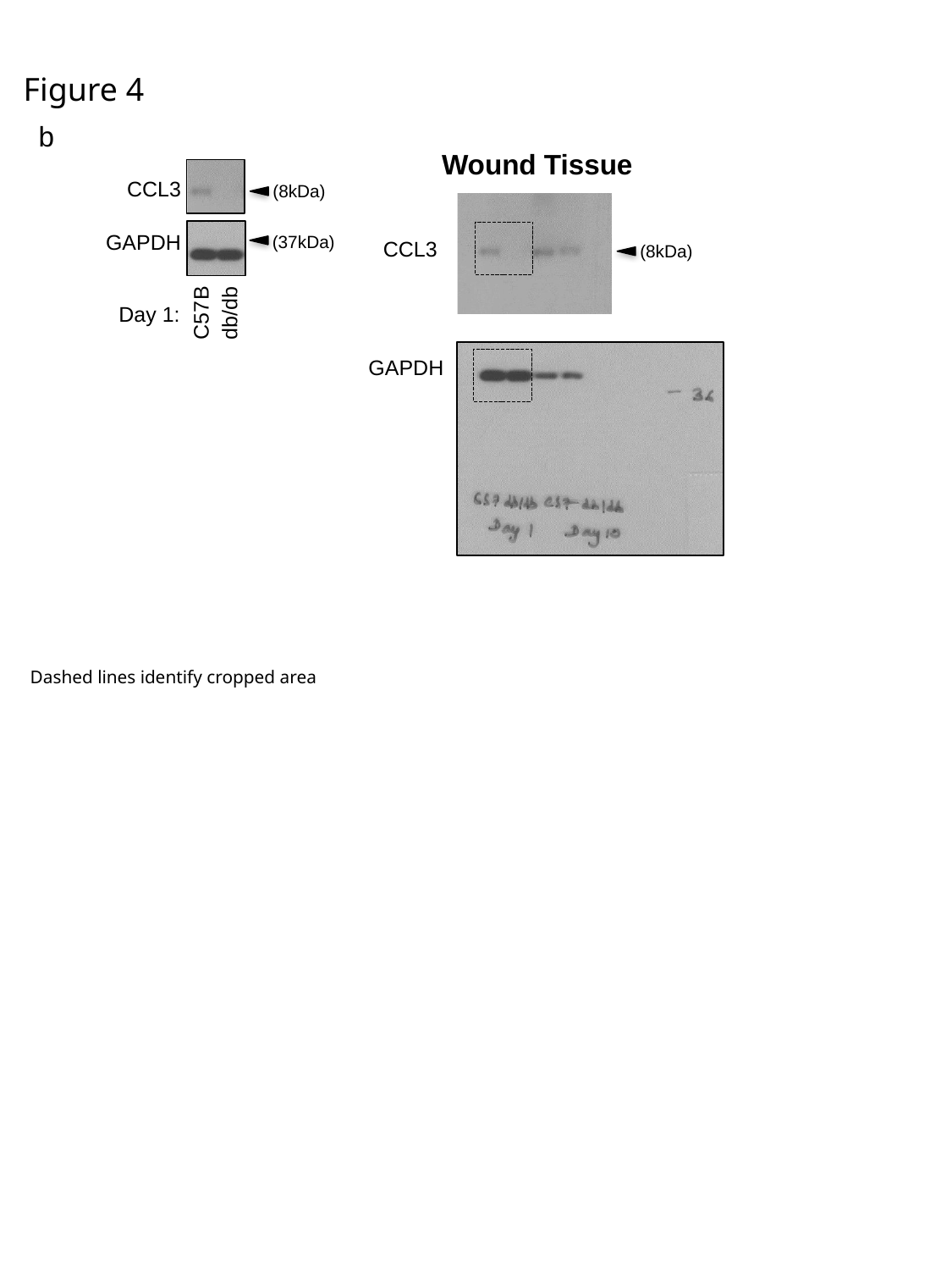

Figure 4
b
Wound Tissue
CCL3
(8kDa)
GAPDH
CCL3
(37kDa)
(8kDa)
db/db
C57B
Day 1:
GAPDH
db/db
C57B
Dashed lines identify cropped area
